# Type-1 Ryanodine Receptor Plays an Important Role in Cardiac Hypertrophy and Heart Failure by Increasing Type-2 Ryanodine Receptor-Mediated Calcium Release

**DOI:** 10.3390/ijms27104291

**Published:** 2026-05-12

**Authors:** Yong-Xiao Wang, Ed Wilson Santos, Sarahann Mistretta, Yuexing Yuan, Harold A. Singer, Shey-Shing Sheu, Yun-Min Zheng

**Affiliations:** 1 Department of Molecular and Cellular Physiology, Albany Medical College, Albany, NY 12208, USA; cavalce@amc.edu (E.W.S.); mistres@amc.edu (S.M.); singerh@amc.edu (H.A.S.); 2 Center for Translational Medicine, Department of Medicine, Sidney Kimmel Medical College, Thomas Jefferson University, Philadelphia, PA 19107, USA; yuexing.yuan@jefferson.edu (Y.Y.); shey-shing.sheu@jefferson.edu (S.-S.S.)

**Keywords:** cardiac hypertrophy, heart failure, RyR1, RyR2, Ca^2+^ release

## Abstract

Type-1 ryanodine receptor (RyR1) is essential for skeletal muscle contraction. This Ca^2+^ release channel is expressed in cardiac myocytes; however, its function remains elusive. Cardiac-specific RyR1 overexpression (OE) mice were generated under the cardiac-specific Myh6 promoter. Cardiac hypertrophy (CH), cardiac functions, and mechanistic changes in RyR1 OE and control (wildtype, WT) mice were assessed using hematoxylin and eosin staining, echocardiography, electrocardiogram, quantitative RT-PCR, Western blotting, [^3^H]-ryanodine binding assay, confocal microscope, ROS dye Amplex Red and 2′,7′-dichlorofluorescein diacetate. RyR1 OE mice had increased whole heart, left ventricular weight, and left ventricular wall thickness, but decreased cardiac output and stroke volume, thereby presenting CH and heart failure (HF). CH markers like ANF, BNF, and aSKA mRNAs were increased in RyR1 OE heart. RyR1, but not RyR2 or RyR3, expression was increased in the RyR1 OE mouse heart. Similar results were found in mice with TAC-induced CH. RyR1, but not RyR2 mRNA, was increased in cardiac muscle from dogs and humans with CH and/or HF. Maximum [^3^H]-ryanodine binding was increased, whereas the binding dissociation constant decreased in left ventricular cardiomyocytes from RyR1 OE mice. RyR2-dependent Ca^2+^ sparks were increased, which was blocked by riluzole, a small molecule known to inhibit RyR2. Consistently, ROS was remarkably increased in RyR1 OE cardiac cells. We first generated cardiac-specific RyR1 OE mice; these mice had CH, HF, and increased RyR1 expression with no RyR2 or RyR3 alteration. Similar changes were observed in mice, dogs, and humans with CH and HF. Increased mitochondrial ROS-dependent RyR2 Ca^2+^ release was essential for RyR1-induced CH and HF.

## 1. Introduction

Ryanodine receptor type 1 (RyR1) is predominantly expressed and important for functions in skeletal muscle, and this channel is also expressed and functional in smooth muscle [1,2]. In cardiac muscle cells, reports have shown that low levels of RyR1 mRNA and/or protein exist in cardiomyocytes of mice, pigs, and humans [3,4,5]. Furthermore, it has been shown that in the failing human heart, expression of RyR1 in left ventricular cardiomyocytes increased by 70% [4,6]. However, the direct functional role of RyR1 in the heart is still mostly unveiled [1].

In contrast to RyR1, RyR2 is the predominant subtype in cardiac muscle and critical for the functions in left ventricular cardiomyocytes; moreover, recent studies have further shown that this Ca^2+^ release channel is substantially expressed and functional in smooth muscle [1,7]. As for the standard excitation-contraction coupling [8,9,10], RyR1 is activated by a direct physical association with the voltage-dependent Ca^2+^ channels (VDCCs) on the cell membrane, causing rapid, massive Ca^2+^ release, whereas RyR2 depends on Ca^2+^-induced Ca^2+^ release (CICR), where a small amount of Ca^2+^ entering via VDCCs triggers a further large Ca^2+^ release from the SR. Apparently, RyR2 is more sensitive to Ca^2+^ than RyR1, allowing for its faster cellular responses. It is also known that calmodulin may inhibit RyR2 at all various concentrations, while having biphasic effects on RyR1. More interestingly, RyR1 mutations lead to skeletal muscle diseases like malignant hyperthermia and central core disease; however, RyR2 mutations cause cardiac diseases such as catecholaminergic polymorphic ventricular tachycardia and even HF.

Noticeably, a global RyR2 knockout (KO) results in embryonic lethality in mice and rabbits [6,11]. To rescue the embryonic lethality of RyR2 KO, we designed a strategy to create cardiac RyR1 overexpression (OE) mice and then breed these OE mice with RyR2 KO mice for rescue. Unfortunately, RyR1 OE mice cannot rescue the embryonic lethality of RyR2 KO mice. To our surprise, our preliminary findings indicated that RyR1 OE mice developed significant cardiac hypertrophy compared with their control (wildtype, WT) animals. These unexpected results suggested that RyR1 might exhibit increased expression and activity, thereby leading to cardiac hypertrophy. Cardiac hypertrophy is one of the major factors for cardiovascular diseases (CVDs), which are the number one cause of death in the US, accounting for one out of every three deaths [12]. Heart hypertrophy can be caused by various factors, including hypertension, hypertrophic cardiomyopathy, obesity, pregnancy, etc. This common and devastating disease often leads to heart failure, arrhythmias, and even sudden cardiac death [13].

Currently, the molecular mechanisms for cardiac hypertrophy are not fully understood; the existing therapies cannot offer a cure and are often not effective in this devastating disease [14,15]. These concerns, with the aforementioned descriptions, inspired us to determine the potentially important role of the poorly understood RyR1 in the development of cardiac hypertrophy and explore the novel signaling pathways for its underlying role, particularly using our recently created RyR1 OE mice.

## 2. Results

### 2.1. RyR1 OE Causes Cardiac Hypertrophy and Heart Dysfunction in Mice

**Figure 1:**
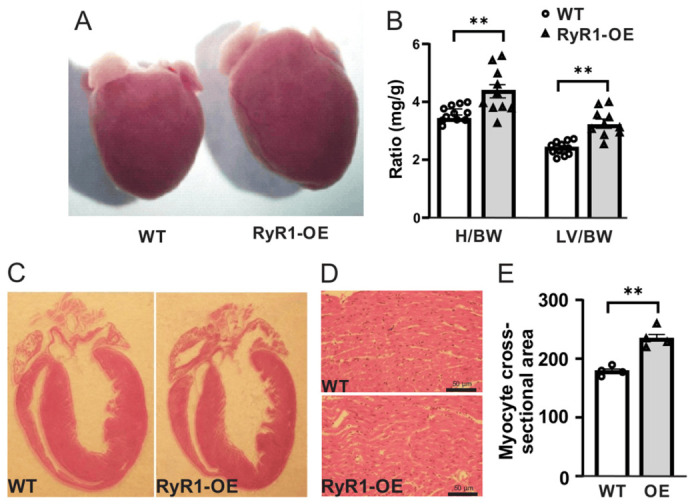
**RyR1 OE resulted in cardiac hypertrophy in mice.** (**A**) The original image showed that the gross whole heart size was largely increased in cardiac RyR1 OE mice compared with wild-type (WT, control) mice. (**B**) Ratio of the whole heart and left ventricle weight was largely increased in RyR1 OE mice. (**C**) Longitudinal view of the heart with H&E staining in WT and RyR1 OE mice. (**D**) Cross-sectional view of the cardiomyocytes on H&E staining. (**E**) The average cross-sectional area of H&E-stained cardiomyocytes was quantified using ImageJ (version 1.54). ** *p* < 0.01 compared with control mice.

As described above, we bred our generated RyR1 OE mice with RyR2 KO mice to rescue the RyR2 KO early embryonic lethality. Although it did not, RyR1 OE only mice developed cardiac hypertrophy. Herein, we sought to continue these preliminary studies for confirmation. As expected, the whole heart size was significantly larger compared with control (wildtype, WT) mice (Figure 1A). Consistent with this result, the ratio of the whole heart and left ventricle weight relative to body weight was largely increased (Figure 1B).

In support of the increased left ventricular weight, we performed histological assessment in WT and RyR1 OE mouse hearts using H&E staining. The results were shown in Figure 1C–E, indicating a larger cardiomyocyte cross-sectional area in RyR1 OE mice compared with controls. These findings further reflect cardiac hypertrophy in RyR1 OE mice.

Echocardiograph analysis also discovered the increased LVPWD, LVPWS, and LVRI, as well as decreased CO and SV (Table 1). These abnormal morphological and functional changes further reveal the cardiac hypertrophy in RyR1 OE mice, as described in Figure 1.

Cardiac hypertrophy often promotes and even causes cardiac arrhythmias in human and animal models [16]. Therefore, we performed ECG rhythm analysis in conscious mice using the ECGenie system eMOUSE/EzCG Analysis Software (https://mousespecifics.com/heart/ecgenie/, accessed on 29 April 2026). As shown in Table 2, the results have disclosed that heart rate (HR) was increased. Moreover, QRSs and the QT interval were significantly longer. All these findings indicate that RyR1 OE mice show a high-risk electrical instability and even develop cardiac arrhythmias.

**Figure 2:**
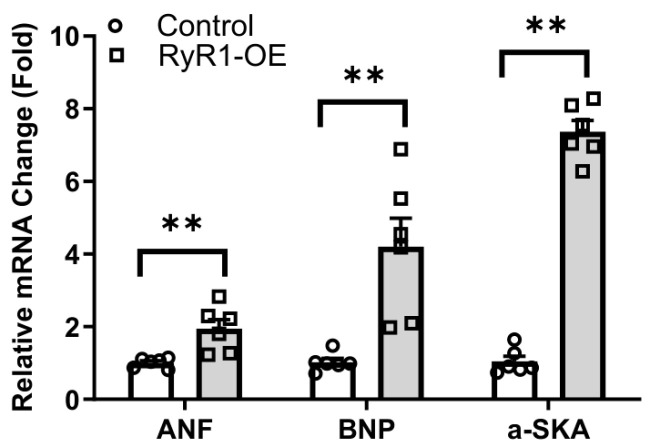
Real-time RT-PCR assay revealed that cardiac hypertrophic marker genes ANF, BNP and αSKA mRNA expression levels were elevated in left ventricular myocardial cells from RyR1 OE mice. Data was obtained from 6 animals in each group. ** *p* < 0.01 compared with control mice.

Interestingly, we have found that the standard and common cardiac hypertrophic marker genes ANF, BNP, and αSKA mRNA expression levels were markedly elevated in cardiac RyR1 OE mice, as presented in Figure 2.

**Figure 3:**
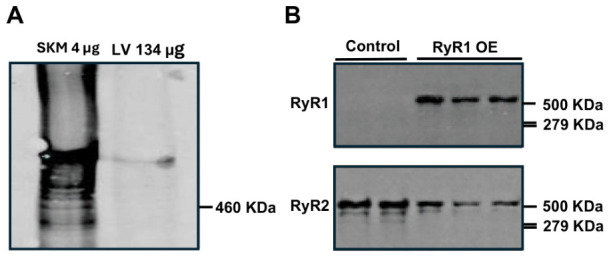
RyR1 protein expression in the mouse heart. (**A**) Gel electrophoresis of Western blotting revealed that left ventricular (LV) muscle, similar to skeletal muscle (SKM), expressed RyR1 protein. (**B**) RyR1 protein expression was significantly increased in LV form RyR1 OE mice compared with control (WT) mice.

With the meaning of Western blotting, we found RyR1, but not RyR2, protein expression level was largely increased in heart muscle from cardiac RyR1 OE mice (Figure 3).

Together, all these data demonstrate that cardiac RyR1 OE evokes cardiac hypertrophy and implicates heart function in mice.

### 2.2. RyR1 mRNA Expression Is Significantly Increased in Cardiac Myocytes from Animals and Human Patients with Various Cardiac Hypertrophy and Heart Failure

**Figure 4:**
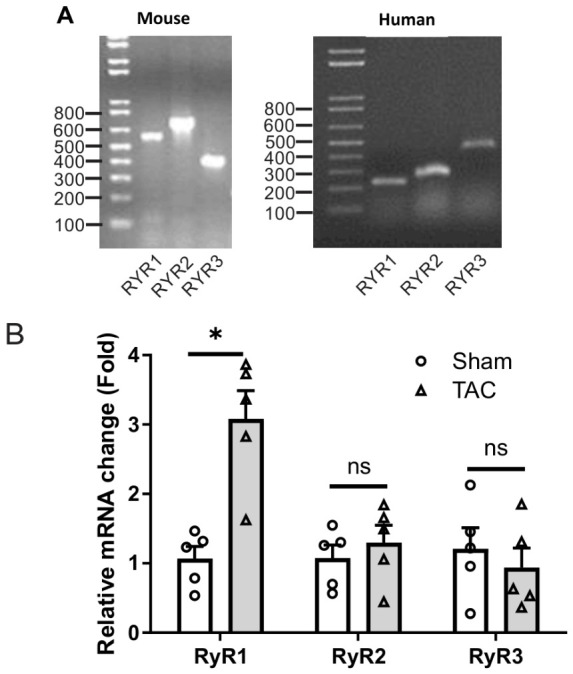
RT-PCR indicated that all RyR1, RyR2 and RyR3 mRNA were expressed in both mouse and human cardiac myocytes. (**A**) Original agarose gel electrophoresis of RT-PCR products in cardiac myocytes from mice (left) and humans (right). (**B**) Real-time RT-PCR indicates that RyR1, neither RyR2 nor RyR3, mRNA expression level was significantly increased in cardiac myocytes from mice following TAC. * *p* < 0.05 compared with sham group (*n* = 5 per group).

**Figure 5:**
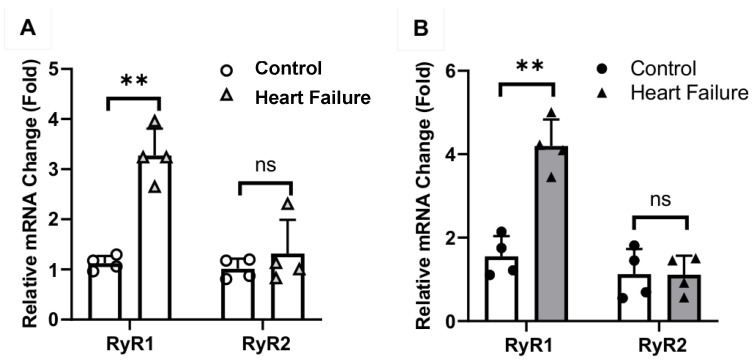
RyR1 mRNA expression was remarkably increased in the hearts of dogs and humans with heart failure. (**A**) RyR1 mRNA expression level was significantly higher in the hearts of dogs with pacing-induced heart failure. In contrast, RyR2 mRNA expression level was not changed. ** *p* < 0.01 compared with control (*n* = 4 per group). (**B**) RyR1, but not RyR2, mRNA levels were largely increased in the hearts of humans with heart failure. ** p < 0.01 compared with control (*n* = 4 per group). ns indicated no statistical difference compared with control.

As we and others reported previously [17,18], TAC was performed in mice to restrict aortic blood flow and thus increase blood pressure, leading to cardiac hypertrophy and heart failure in the later step. Using the quantitative RT-PCR as we described previously [19,20], we found that RyR1, RyR2, and RyR3 all showed a significant mRNA expression in left ventricular myocytes from mice and human subjects, as shown in agarose gel electrophoresis (Figure 4A). More importantly, we observed that RyR1 mRNA, but not RyR2 or RyR3 mRNA, was much higher in expression level in cardiac myocytes from mice following TAC for 3 weeks compared with control mice (Figure 4B). Similarly, RyR1 mRNA expression level was largely increased in the heart from dogs with pacing-induced HF; in contrast, RyR2 mRNA expression level was unaffected (Figure 5A). Moreover, we further discovered the increased mRNA expression level of RyR1 with no change in RyR2 mRNA expression in the hearts of heart failure patients. All these results demonstrate that RyR1 may mediate cardiac hypertrophy and heart failure.

### 2.3. RyR1 OE Causes an Apparent Increase in RyR Activity in Mice

**Figure 6:**
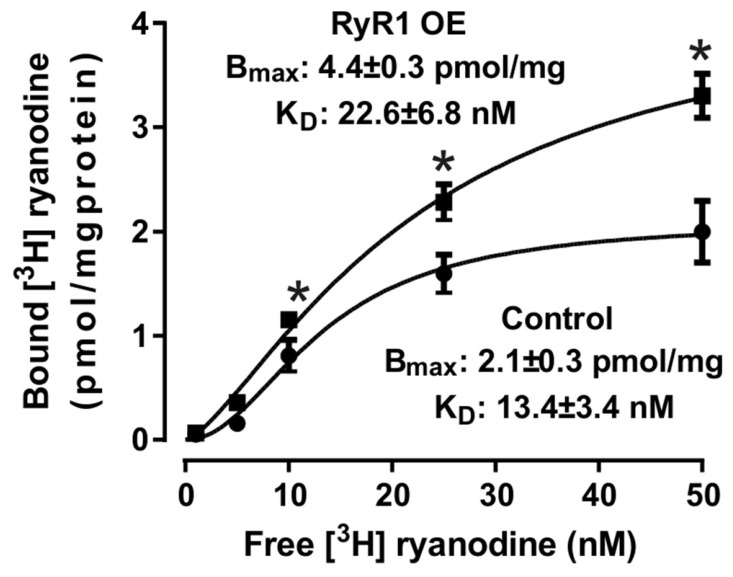
The maximal [^3^H]-ryanodine binding (Bmax) is largely increased, whereas the binding dissociation constant (KD) decreased, in isolated left ventricular mitochondrial fractions from RyR1 OE mice and control mice. * *p* < 0.05 compared with control mice.

It is well known that the [^3^H]-ryanodine binding assay is a common and standard method to determine the activity of RyR in cardiovascular muscle cells [21]. Using this method, we observed that the maximum [^3^H]-ryanodine binding was greatly increased, whereas the binding dissociation constant decreased in RyR1 OE left ventricular cardiomyocytes (Figure 6).

**Figure 7:**
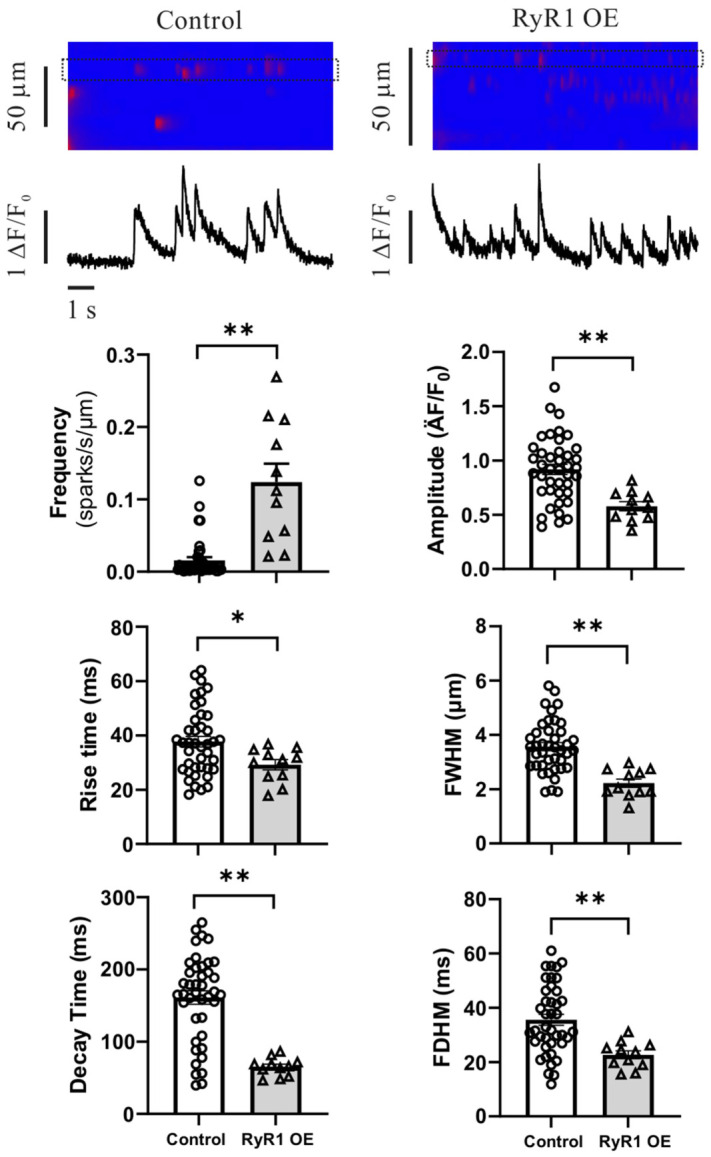
Ca^2+^ sparks are largely increased in isolated left ventricular myocardial cells from RyR1 OE mice. Cells were loaded with fluo4/AM. Ca^2+^ sparks were recorded using a Zeiss laser scanning confocal microscope with a line-scanning mode. Data were obtained from cells of 4 control mice and 3 RyR1 OE mice. * *p* < 0.05, ** *p* < 0.01 compared with control.

In support of this increased RyR activity, our further studies using the laser scanning confocal microscope (Zeiss, Jena, Germany) unveiled that Ca^2+^ spark activity was significantly increased, although the amplitude slightly decreased in freshly isolated left ventricular cardiomyocytes from adult cardiac RyR1 OE mice (Figure 7).

### 2.4. RyR2 May Mediate the Increased RyR Activity in Cardiac Myocytes from Cardiac RyR1 OE Mice

**Figure 8:**
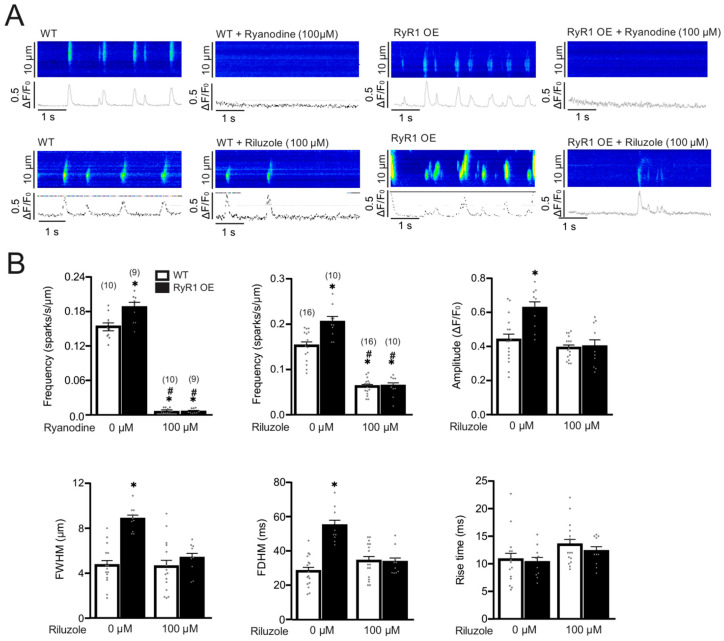
RyR2 may mediate the increased Ca^2+^ sparks in isolated neonatal cardiac myocytes from RyR1 OE mice. (**A**) Original recordings of Ca^2+^ sparks in WT and RyR1 OE mouse left ventricular cardiomyocytes before and after treatment with ryanodine or riluzole for 10 min. Cells were loaded with fluo4/AM, and Ca^2+^ sparks were recorded using a Zeiss laser scanning confocal microscope with a line-scanning mode. (**B**) Summarized effects of ryanodine and riluzole on Ca^2+^ sparks. Numbers in parentheses indicate the number of cells from 8 control mice and 5 RyR1 OE mice. * *p* < 0.05 compared with control. # *p* < 0.05 compared with RyR1 OE.

It is generally believed that the Ca^2+^ spark is generated due to the opening of a cluster of RyR2 in cardiac cells [22]. As such, we sought to investigate whether the increased Ca^2+^ spark activity is mediated by RyR2 in neonatal cardiac myocytes from cardiac RyR1 OE mice. A recent report has shown that riluzole is a small molecule that inhibits RyR2 only, not RyR1 and RyR3 in cardiac cells. Thus, this molecule was selected to determine its effect. As presented in Figure 8, consistent with the role of RyR2, treatment with riluzole almost fully inhibited the increased activity of Ca^2+^ sparks in freshly isolated cardiac myocytes from cardiac RyR1 OE mice.

### 2.5. The Role of RyR2 Is Likely to Be a Result of the Increased Mitochondrial ROS in Cardiomyocyte from RyR1 OE Mice

**Figure 9:**
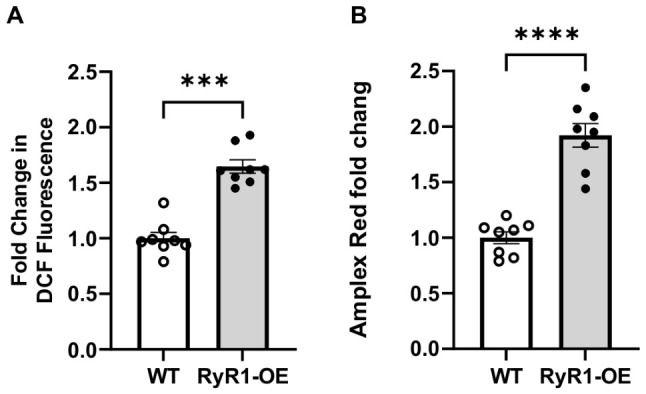
Mitochondrial ROS are significantly increased in the hearts of RyR1 OE mice. ROS production is determined by measuring the fluorescence of the ROS detection dye DCF (**A**) and Amplex Red (**B**) in isolated mitochondria from control and RyR1-OE mice. *** *p* < 0.001, **** *p* < 0.0001 compared with the control group.

It is known that RyR2 is highly reactive to ROS; thus, we wondered whether ROS could be increased in RyR1 OE left ventricular cardiomyocytes, thereby leading to increased RyR2 activity. Using the common and cell-permeant fluorescent dye 2′,7′-dichlorofluorescin diacetate (DCFDA, also known as H_2_DCFDA) to measure ROS, including superoxide (O_2_^−^) and hydrogen peroxide (H_2_O_2_) [23,24], we unveiled that DCF-derived fluorescence was largely increased in isolated mitochondria from cardiac myocytes of cardiac RyR1 OE mice compared with WT mice (Figure 9A). In support, using Amplex UltraRed fluorescence to detect H_2_O_2_ has largely increased as well (Figure 9B). These results indicate that the role of RyR2 is likely to be a result of increased mitochondrial ROS in cardiomyocyte mitochondria from RyR1 OE mice.

## 3. Discussion

Increasing evidence reveals that RyR1 mRNA and/or protein are expressed in cardiomyocytes of mice, dogs, pigs, and humans [3,4,5]. Furthermore, in the failing human heart, expression of RyR1 in left ventricular cardiomyocytes is increased by 70% [4,6]. These data suggest that this channel may be involved in cardiac disease(s). RyR2 KO mice die at an early embryonic stage [11]. Thus, we sought to generate RyR1 OE mice and then bred these mice with RyR2 KO mice for salvage. Unlike our prediction, RyR1 OE mice did not rescue the early embryonic lethality of RyR2 KO mice. To our surprise, however, our preliminary investigations indicated that RyR1 OE developed cardiac hypertrophy with damaged heart function. A number of further studies described herein confirmed that these OE mice showed a larger overall gross heart size as well as increased heart and left ventricle weight (Figure 1). Moreover, these RyR1 OE mice had increased LVPWD, LVPWS, and LVRI, whereas they had decreased CO and SV (Table 1). We also found that the RyR1 OE mice presented largely increased RyR1 protein expression levels (Figure 2). In support, we have found that the cardiac hypertrophic marker genes ANF, BNP, and αSKA mRNA expression levels were substantially increased in left ventricular cardiomyocytes from RyR1 OE mice (Figure 3).

Similar to RyR1 OE, TAC caused not only cardiac hypertrophy and heart failure, but also increased RyR1 expression (Figure 4). The increased RyR1 expression levels were further revealed in various animals, including dogs and humans with cardiac hypertrophy and heart failure (Figure 5).

Cardiac hypertrophy is a major factor for heart failure and other cardiovascular diseases (CVDs), which are the number one cause of death in the US, accounting for 1 out of every 3 deaths [12]. This disease can be caused by various factors, including high blood pressure, hypertrophic cardiomyopathy, pregnancy, etc. This common and devastating disease often leads to heart failure, arrhythmias, and even sudden cardiac death [13]. Currently, the molecular mechanisms for cardiac hypertrophy are not fully understood; the existing therapies cannot offer a cure and are often not effective in this devastating disease [14,15]. Our current findings not only demonstrate that RyR1 may play an important role in the development of cardiac hypertrophy and heart failure, but also suggest that specific targets at RyR1 provide a novel and effective therapeutic intervention for cardiac hypertrophy and heart failure.

It is known that increased intracellular Ca^2+^ signaling is a major factor in the development of cardiac hypertrophy [25,26,27]. In agreement with this well-accepted viewpoint, we have found that the activity of RyRs, determined by using [^3^H]-ryanodine binding assay, is largely increased in isolated mitochondrial fractions of left ventricular cardiomyocytes from RyR1 OE mice (Figure 6). In support, the activity of Ca^2+^ sparks is largely increased in left ventricular cardiomyocytes from RyR1 OE mice as well (Figure 7).

In cardiac myocytes, Ca^2+^ sparks are mainly generated by Ca^2+^ release from a cluster of RyRs [28,29]. Thus, we were wondering whether the increased activity of Ca^2+^ sparks is mediated by RyR2 in left ventricular cardiomyocytes from RyR1 OE mice. A recent RyR2 inhibitor screening using a well-characterized compound library with a newly developed SR Ca^2+^-based assay has found that riluzole, a small molecule drug used to clinically treat amyotrophic lateral sclerosis, is a novel, specific, and potent inhibitor of RyR2, but not RyR1 or RyR3 [30]. Interestingly, this novel RyR2 inhibitor blocked the increased Ca^2+^ sparks in left ventricular cardiomyocytes from RyR1 OE mice (Figure 8). It is generally accepted that RyR2 is the only subtype of the three RyRs on the SR. Taken together, increased intracellular Ca^2+^ is mediated by RyR2 in left ventricular cardiomyocytes of RyR1 OE mice.

Our preliminary studies indicated that RyR1 OE was unable to rescue the embryonic lethality of RyR2 complete KO mice. This phenomenon is consistent with our previous reports and others that RyR1 is not molecularly and functionally expressed on the SR of cardiac myocytes, but rather on mitochondria [31,32,33,34,35]. A similar finding has been observed in neurons [36]. It is worth pointing out that RyR2 exhibits greater Ca^2+^ sensitivity and a higher density of localization on the SR than RyR1 [8,9,10]. Moreover, a previous report has shown that heterozygous loss-of-function RyR2 leads to catecholamine release deficiency syndrome [37]. Apparently, it would be interesting to check the phenotypes in RyR1 OE mice after heterozygous loss of one RyR2 allele and the effect of riluzole in a cell line with a RyR2 gain-of-function mutation. However, these two subjects not only fall outside our current scope, but are also reserved for future research.

It is widely accepted that RyR2 is highly reactive to ROS in all types of cells, including cardiac myocytes and smooth muscle cells [29,38]. Thus, we were wondering whether ROS could be increased in RyR1 OE left ventricular cardiomyocytes, thereby leading to increased RyR2 activity. Using the DCF fluorescence ROS probe [23,24], we have found that the substantially higher H_2_O_2_ production in isolated mitochondria from left ventricular cardiomyocytes of RyR1 OE mice (Figure 9). Apparently, the increased activity of RyR2 is likely to be a result of increased mitochondrial ROS in cardiomyocytes from RyR1 OE mice.

In conclusion, our current findings demonstrate that cardiac-specific RyR1 OE mice develop cardiac hypertrophy and heart failure. These mice also show much higher RyR1, but not RyR2 and RyR3, mRNA and protein expression levels in left ventricular cardiomyocytes. We have also found that TAC in mice not only causes cardiac hypertrophy and heart failure, but also substantially increases RyR1 mRNA and protein expression with no change in RyR2 and RyR3 expression in left ventricular cardiomyocytes. In support, only RyR1 mRNA expression levels are significantly higher in left ventricular cardiomyocytes from dogs and humans with cardiac hypertrophy and heart failure. Moreover, the activity of RyR2 is largely increased to induce more Ca^2+^ release in left ventricular myocytes from RyR1 OE mice. Finally, we have discovered that the increased RyR2 activity is likely to be a result of the increased mitochondrial ROS production in left ventricular myocytes from RyR1 OE mice. Together, the current novel findings also suggest that specific RyR1 targets may become an innovative therapeutic to treat cardiac hypertrophy and heart failure.

## 4. Translational Perspective

Our study for the first time reveals that cardiac-specific RyR1 OE mice develop significant CH and HF. Similarly, RyR1 plays an essential role in mediating CH and HF in other animals and humans. The key role of RyR1 may result from the increased RyR2 Ca^2+^ release. The increased RyR2 activity occurs due to the augmented mitochondrial ROS. Moreover, our findings also suggest that specific genetic and pharmacological inhibition of RyR1 can be a novel and effective approach for targeted therapy in patients with CH and HF.

## 5. Materials and Methods

### 5.1. Generation of Cardiac RyR1 Overexpression (OE) Mice

To generate cardiac-specific RyR1 transgenic mice, the plasmid was constructed by the α-myosin heavy chain (αMHC) promoter (provided by Dr. Jeffery Robbins, Children’s Hospital Medical Center, Cincinnati, OH, USA) driven by RyR1 full-length DNA (provided by Dr. Paul D. Allen, Brigham and Women’s Hospital, Boston, MA, USA), followed by SV40 PolyA. The resulting recombinant plasmid was confirmed by restriction mapping and nucleotide sequencing. The purified plasmid was microinjected into C57BL/6 zygotes and implanted into the oviducts of SJL pseudo-pregnant female mice. The resulting C57BL/6 X SJL F1 pups were subsequently screened by diagnostic PCR and backbred to C57BL/6J mice for at least 5 generations before use. All animal protocols were approved by the Institutional Animal Care and Use Committee of Albany Medical College.

### 5.2. Histologic Assessment of Cardiomyocyte Size

Histological staining of mouse heart tissue was performed to further evaluate cardiac hypertrophy using hematoxylin and eosin (H&E) staining, as described previously [39]. After standard processing, 5 μm sections were stained with H&E to assess increases in cardiomyocyte cross-sectional area by sufficiently defining adequate cellular margins, especially in cross-sectioned cardiomyocytes, where nuclear, cytoplasmic, and surrounding connective tissue were present.

### 5.3. Electrocardiogram

High-risk features of cardiac arrhythmias frequently occur in both human and animal models with cardiac hypertrophy [16]. Accordingly, cardiac rhythm was conducted in conscious mice by using an electrocardiogram (ECG) with the ECGenie System (Version 2.09) for acquisition and eMOUSE/EzCG Analysis Software (Version 17, Mouse Specifics Inc., Framingham, MA, USA). Data were collected and processed to obtain primary ECG parameters including heart rate (HR), HR variability (HRV), HR coefficient of variation (CV), RR, PQ, PR, QRS, QT, ST, corrected QT, QT dispersion, QTc dispersion interval, mean sinus rhythm (SR) amplitude, mean R amplitude, root mean square of successive differences (rMSSD), and vasovagal tonus index (VVTI) to determine arrhythmia incidence.

### 5.4. Echocardiography

Mice were anesthetized with 2% isoflurane in oxygen, after being shaved of hair over the chest region of interest, applied with ultrasound gel for a coupling medium, and underwent echocardiography using the VisualSonics Vevo 3100 system (FUJIFILM VisualSonics, Toronto, ON, Canada) equipped with a probe (MS-550D). The data were analyzed to determine cardiac function.

### 5.5. Quantitative RT-PCR

In these experiments, total RNAs were isolated from cardiac muscle, as we reported previously [19]. The first-strand cDNAs were synthesized from RNAs. The resultant cDNAs were amplified by specific forward and reverse primers for RyR1, RyR2 and RyR3 using the Real-Time PCR Detection System (Bio-Rad, Hercules, CA, USA). The forward and reverse primers were: 5′-CCGGCGATGAATATGAACTT-3′ and 5′-TGATAGCCAGCAGAATGACG-3′ for the mouse RyR1 gene; 5′-CATGGACAGCTTCCCCTGAA-3′ and 5′-GTGTGACTGCCGTGCTTGG-3′ for the mouse RyR2 gene; 5′-CTGGCCATCATTCAAGGTCT-3′ and 5′-GTCTCCATGTCTTCCCGTA-3′ for the mouse RyR3 gene. 5′-CCTGACTGAAGACCACAGTTT-3′, 5′-GCTTCTTCTCCTCCACTTCTTC-3′ for the dog RyR1 gene; 5′-CTCCCGGTCTTCAACTGATAAA-3′, 5′-GCTTAGAGGCAGGGTGAATAG-3′ for the dog RyR2 gene; 5′-CCTGACTGAAGACCACAGTTT-3′, 5′-GCTTCTTCTCTTCCACCTCTTC-3′ for the human RyR1 gene; 5′-CTGAGGAAGACCACCTGAAAG-3′, 5′-AAGAGAGGGTAGAAGGCATAGA-3′ for the human RyR2 gene.

In experiments to test ANF, BNP, and αSKA mRNA expressions, their forward and reverse primers were, respectively, Forward 5′-GGGGGTAGGATTGACAGGAT-3′, Reverse 5′-GCAGAA TCGACTGCCTTTTC-3′; Forward 5′-GCCAGTCTCCAGAGCAATTC-3′, Reverse 5′-CCTTGG TCCTTCAAGAGCTG-3′ and Forward 5′- GTCCACCTTCCAGCAGATGT-3′, reverse 5′-TTG TCGATTGTCGTCCTGAG-3′.

### 5.6. Western Blotting

This blot analysis was performed, as we described before [19,20]. Sample proteins were separated using the standard SDS-PAGE procedure, transferred to a polyvinylidene fluoride membrane (Bio-Rad, Hercules, CA, USA), and then incubated with primary antibodies followed by horseradish peroxidase-conjugated secondary antibodies. Blots were visualized by using an enhanced chemiluminescence (ECL) kit (Santa Cruz, Dallas, TX, USA) and analyzed by Multi Gauge software version 3.0 (Fujifilm Science Systems, Tokyo, Japan).

### 5.7. Transverse Aortic Constriction (TAC)

TAC was performed according to the procedure as described previously [17,18]. Briefly, mice were anesthetized with an intraperitoneal injection of avertin (250 mg/kg), as this anesthetic has a minimal cardiovascular effect, followed by hair removal on the surgical site and then the site disinfection with alcohol. After that, an incision was made on the skin and trachea to insert a 19-gauge cannula and connect it to a small animal ventilator for ventilation. After disinfection, a thoracotomy incision was made to expose the ascending aorta, the aorta ligated with a blunt 26-gauge needle using a silk suture, and then the needle was removed to cause aortic banding (stenosis), which increases blood flow resistance. The air/fluid in the thoracic cavity was evacuated, the thoracic cavity regained the normal negative physiologic intrathoracic pressure, the intercostal muscles, ribs, and skin incision were closed, and the animals were allowed to recover from anesthesia. As control, mice received a pseudo-surgery, in which the same surgery was performed, but no silk suture was used to cause aortic banding.

### 5.8. Ca^2+^ Spark Measurement

The measurement of Ca^2+^ sparks was made in freshly isolated left ventricular myocytes using the Zeiss LSM 510 or LSM880 laser scanning (Zeiss, Jena, Germany) confocal microscope in a line-scan mode [22,40]. To obtain isolated myocytes, hearts were cannulated on a Langendorff perfusion apparatus and perfused with a modified KH (Krebs–Henseleit) solution for 15 min, then with the Ca^2+^-free KH solution for 4 min, and finally digested with the modified KH solution containing collagenase, protease and trypsin for ~10 min. After stopping digestion with bovine serum albumin added in a modified KH buffer, the left ventricular tissue will be removed, dispersed, and filtered. After gradual Ca^2+^ reintroduction, isolated myocytes were loaded with fluo-4/AM. The dye was excited at 488 nm, and the emission fluorescence was detected at 510 nm. Ca^2+^ spark frequency, amplitude, and other spatiotemporal parameters were analyzed using custom-made software written in Interactive Data Language [22,40].

### 5.9. [^3^H]-Ryanodine Binding Assay

This binding assay was conducted, as we described previously [21]. Isolated sarcoplasmic reticulum (SR) and mitochondrial fractions were prepared from left ventricles and incubated with [^3^H]-ryanodine at 5–25 nM. The radioactivity was determined by liquid scintillation counting. Nonspecific binding was assessed with unlabeled ryanodine.

### 5.10. Mitochondrial Reactive Oxygen Species (ROS) Measurement

Isolated mitochondria were obtained from left ventricular myocytes of mice using a differential centrifugation method, as we reported previously [41]. The isolated mitochondria were added to microplate wells containing respiration buffer with 2′,7′-dichlorofluorescein diacetate (DCF/DA) and horseradish peroxidase to mainly measure H_2_O_2_ [23,24]. DCF-derived fluorescence was measured using the FlexStation-3 spectrophotometer (Molecular Devices) with an excitation wavelength of 485 nm and emission wavelength of 532 nm. Amplex UltraRed was also used to detect H_2_O_2_ with an excitation wavelength of 530 nm and emission wavelength of 590 nm.

### 5.11. Statistics

All statistical data were expressed as means ± S.E. of *n* experiments investigated. The Student’s *t*-test and one- or multi-way ANOVA with appropriate post hoc analysis were used to determine the significance of differences between comparisons. *p* < 0.05 was accepted as a statistically significant level.

## Figures and Tables

**Table 1:** Echocardiographic analysis revealed cardiac hypertrophy in cardiac RyR1 OE mice. The results revealed the increased LVPWD, LVPWS, and LVRI, and decreased CO and SV. * *p* < 0.05 compared with control (wildtype, WT) mice.

Parameters	WT (*n* = 9)	RyR1 OE (*n* = 8)
IVSD (mm)	0.85 ± 0.09	0.97 ± 0.14
IVSS (mm)	1.38 ± 0.21	1.45 ± 0.25
LVIDD (mm)	3.97 ± 0.22	3.58 ± 0.57
LVIDS (mm)	2.68 ± 0.22	2.33 ± 0.55
LVPWD (mm)	0.81 ± 0.13	1.14 ± 0.42 *
LVPWS (mm)	1.19 ± 0.16	1.52 ± 0.44 *
EF (%)	65.07 ± 3.74	63.01 ± 9.75
FS (%)	35.23 ± 2.83	34.03 ± 6.96
CO (mL/min)	20.49 ± 3.61	15.21 ± 4.39 *
SV(μl)	45.95 ± 7.11	30.5 ± 13.01 *
RWT	0.41 ± 0.07	0.56 ± 0.21
LVRI	30.82 ± 3.96	40.77 ± 6.22

IVSD, interventricular septum diastolic thickness; IVSS, interventricular septum systolic thickness; LVIDD, left ventricular internal diameter end diastole; LVIDS, Left ventricular internal diameter end systole; LVPWD, left ventricular posterior wall diastolic thickness; LVPWS, left ventricular posterior wall systolic thickness; EF, ejection fraction; FS, fractional shortening; CO, cardiac output; SV, Stroke volume; RWT, relative wall thickness; LVRI, left ventricular remodeling index.

**Table 2:** Electrocardiogram (ECG) in conscious WT and RyR1 OE mice using the ECGenie System eMOUSE/EzCG Analysis Software.

Parameter	Unit	WT (*n* = 11)	RyR1 OE (*n* = 10)	*p* Value
HR	bpm	705.8 ± 41.8	737.5 ± 13.1	0.02
HRV	bpm	5.87 ± 1.87	5.98 ± 1.87	0.89
CV	%	1.12 ± 0.55	0.82 ± 0.24	0.1
RR	ms	84.62 ± 5.7	83.07 ± 4.8	0.47
PQ	ms	21.38 ± 3.1	19.80 ± 2.0	0.15
PR	ms	27.34 ± 3.3	25.33 ± 2.0	0.08
QRS	ms	9.30 ± 0.8	11.00 ± 1.9	0.01
QT	ms	40.93 ± 1.6	43.59 ± 2.9	0.01
ST	ms	32.93 ± 2.0	32.13 ± 1.5	0.27
QTC	ms	46.34 ± 2.4	45.35 ± 1.4	0.24
QT dispersion	ms	24.98 ± 3.2	23.36 ± 2.1	0.16
QTc dispersion	ms	27.21 ± 3.6	25.74 ± 2.3	0.25
Mean SR amplitude	mV	0.95 ± 0.32	0.83 ± 0.24	0.3
Mean R amplitude	mV	0.87 ± 0.23	0.72 ± 0.24	0.12
rMSSD	ms	1.17 ± 0.88	0.79 ± 0.13	0.15
VVTI	—	−0.40 ± 0.84	−0.96 ± 0.50	0.06

Data are presented as mean ± SEM.

## Data Availability

The main data presented in this study are subject to privacy law and available on request from the corresponding authors.
